# Assessing right ventricular function in patients with pulmonary hypertension based on noninvasive measurements: correlation between cardiac MRI, ultrasonic cardiogram, multidetector CT and right heart catheterization

**DOI:** 10.1186/1532-429X-17-S1-P185

**Published:** 2015-02-03

**Authors:** Xiaojuan Guo, Min Liu, Zhanhong Ma, tao jiang, Yuanhua Yang, zhenguo zhai, Renyou zhai, Tianjing Zhang

**Affiliations:** Department of Radiology, Beijing Chao-Yang Hospital, Capital Medical University, Beijing, China; Respiratory Diseases Researching Center of Capital Medical University, Beijing, China; Siemens Ltd., China, Beijing, China

## Background

The assessment of right ventricular function is of great importance in the management of patients with pulmonary hypertension (PH). Our aim is to compare the value of cardiac MRI (CMRI), ultrasonic cardiogram (CUS), multidetector CT (MDCT) in assessing right ventricular function, and to evaluate the correlation between parameters derived by MRI, CUS, MDCT and the indicators of right ventricular function derived by right heart catheterization (RHC) in patients with PH.

## Methods

Thirty one consecutive patients with PH (17 males and 14 females; mean age, 54.61±11.74 years; range, 33 to 76 years) were prospectively enrolled.All patients underwent RCH to get hemodynamic parameters .Then, calculations including pulmonary vascular resistance index (PVRI), right cardiac work index (RCWI), right ventricular stroke work index (RVSWI) were performed. All patients underwent CMRI to get parameters including right ventricular end-systolic volume (ESV), end-diastolic dimension (EDV), stroke volume (SV), ejection fraction (EF), the cardiac muscle mass (CMS). 28 patients underwent CUS to get these parameters including right ventricular Tei index, right ventricular fractional area change (RV FAC), RV-ESV, RV-EDV. And 25 patients underwent MDCT to get these parameters including right /left ventricular internal diameter (RVd/LVd), right /left ventricular diastole maximum area (RVa/LVa), cobb angle, right ventricular free wall thickness (RVWT). All examinations were executed within 7days. These parameters obtained by MRI, CUS and MDCT were correlated with those of RCH respectively by Spearman or Pearson correlation analysis.

## Results

Most parameters of RV function derived by CMRI, CUS, MDCT correlated moderately with mPAP, PVRI. mPAP had strong correlation with MRI-ventricular mass index (VMI)(*r*=0.528, *P*=0.002), RVWT (*r*=0.554, *P*=0.005); PVRI had strong correlation with MRI-EF(*r*=-0.647, *P*=0.000), MRI-VMI(*r*=-0.567, *P*=0.001), CT-RVd(*r*=0.536, *P*=0.006), RVd/LVd (*r*=0.530, *P*=0.006), CT-Cobb angle(*r*=0.608, *P*=0.001), RVWT(*r*=0.586, *P*=0.003). Table 1 showed the correlation of right ventricular function between CMRI, CUS, MDCT and RHC. RHC-SV had the strong correlation with MRI-EF(*r*=0.557, *P*=0.001), CT-RVd(*r*=-0.502, *P*=0.011), CT-Cobb angle(*r*=-0.503, *P*=0.003).

**Table 1:** Correlation coefficients of right ventricular function parameters between CMRI, CUS,M DCT and RHC.

	6MWD		RHC-SV		RCWI		RVSWI	
	r	P	r	P	r	P	r	P
MRI-SV(n=31)	0.073	0.713	0.457	0.010	0.322	0.077	0.324	0.075
MRI-EF	0.473	0.011	0.557	0.001	0.389	0.031	0.307	0.093
MRI-VMI	-0.472	0.011	-0.444	0.012	-0.259	0.159	-0.173	0.351
US-SV(n=28)	-0.268	0.186	0.016	0.937	-0.201	0.305	-0.071	0.718
US-EF	-0.019	0.923	0.406	0.026	0.015	0.939	0.023	0.905
US-Tei	-0.175	0.448	-0.480	0.024	-0.387	0.075	-0.419	0.042
US-RVFAC	0.046	0.822	0.385	0.043	0.289	0.136	0.199	0.311
CT-RVd(n=25)	-0.373	0.079	-0.502	0.011	-0.486	0.014	-0.468	0.018
CT-RVa	-0.351	0.109	-0.488	0.016	-0.342	0.102	-0.362	0.082
CT-Cobb angle	-0.569	0.004	-0.503	0.003	-0.430	0.025	-0.395	0.041
CT-RVWT	-0.098	0.666	-0.279	0.186	-0.024	0.911	0.023	0.914

6MWD, 6-min walking distance; EF, ejection fraction; VMI, ventricular mass index; RCWI, right cadiac work index; RHC, right heart catheterization; RVa, right ventricular diastole maximum area; RVd, right ventricular internal diameter; RVFAC, right ventricular fractional area change; RVSWI, right ventricular stroke work index; RVWT, right ventricular free wall thickness; SV, stroke volume; Tei, Tie index; P<0.05.

## Conclusions

The parameters obtained by MRI, CUS and MDCT had moderate correlation with RHC-SV. MRI-EF was the best parameter to reflect RV function which could be used to exactly evaluate RV function in patient with PH. Among these noninvasive measurements, CMRI is the optimal method to assess RV function, and then is the MDCT and the last is CUS.

## Funding

the National 12th Five-Year Researching Foundation (2011BAI11B17).

